# HUC-MSC-derived exosomal miR-16-5p attenuates inflammation via dual suppression of M1 macrophage polarization and Th1 differentiation

**DOI:** 10.1016/j.bbrep.2025.102078

**Published:** 2025-06-09

**Authors:** Yuanjing Zheng, Yue Li, Zhengyang Wei, Yang Wang, Yuanlin Liu, Fengsong Liu, Xue Li, Yi Zhang

**Affiliations:** a Key Laboratory of Zoological Systematics and Application, College of Life Sciences, Hebei University, Baoding, 071002, China; b Beijing Institute of Radiation Medicine, Beijing, 100850, China; c Department of Prosthodontics, Peking University School and Hospital of Stomatology, National Center for Stomatology, National Clinical Research Center for Oral Diseases, National Engineering Research Center of Oral Biomaterials and Digital Medical Devices & Beijing Key Laboratory of Digital Stomatology, NHC Key Laboratory of Digital Stomatology, NMPA Key Laboratory for Dental Materials, Beijing, 100081, China

**Keywords:** Mesenchymal stem cell-derived exosome, Mesenchymal stem cell, Exosomal miRNAs, Cell-free therapy, Production consistency of exosomal miRNAs, Th1 cell differentiation, M1 macrophage polarization

## Abstract

Nowadays mesenchymal stem cell-derived exosomes (MSC-Exos) have emerged as a promising cell-free therapeutic alternative to MSC-based therapies, demonstrating efficacy in treating degenerative diseases, inflammatory disorders, and autoimmune diseases. MSC-Exos transport bioactive cargoes such as proteins, lipids, mRNAs, and microRNAs (miRNAs) to the recipient cells, mediating intercellular communication to regulate immunomodulation and tissue repair. However, the exosomal miRNA profile varies dynamically based on the culture conditions and tissue sources. Thus, elucidating the specific exosomal miRNA profile and regulatory targets is critical for the precise clinical applications and development of MSC-Exos-based cell-free therapies.

Here we established an optimized serum-free culture system for human umbilical cord-derived MSCs (hUC-MSCs) and determined the critical 48–72-h harvest window for exosome secretion. High-throughput sequencing identified miR-16-5p as the predominant exosomal miRNA, functioning as a core immunosuppressive effector by suppressing LPS/IFN-γ-induced M1 macrophage polarization and Th1 cell differentiation. Mechanistically, miR-16-5p was found to target key nodes in NF-κB and JAK-STAT pathways, validated via dual-luciferase assays. Additionally, miR-125b-5p and miR-34a-5p enhanced this immunosuppressive effect by co-targeting overlapping pathway components in NF-κB and JAK-STAT pathways, suggesting a multilayered regulatory network. Taken together, our findings highlight the potential of miRNA-engineered exosomes as standardized therapies for inflammatory disorders, emphasizing the importance of optimizing culture conditions and profiling miRNA expression over time in advancing clinical translation.

## Highlights

• HUC-MSC-derived exosomes were detected in conditioned supernatants, with peak secretion occurring at 48–72 hours.
• High-throughput sequencing identified miR-16–5p as the key exosomal miRNA.
• MiR-16–5p inhibits M1 macrophage polarization and Th1 differentiation by targeting the NF-κB and JAK-STAT pathways.

## 1 Introduction

The immunomodulatory [1] and tissue-regenerative properties [[2], [3], [4]] of mesenchymal stem cells (MSCs) have established them as a cornerstone of cell therapy research. Preclinical and clinical studies highlight their efficacy in treating autoimmune diseases, inflammatory conditions, and degenerative disorders. However, clinical translation of MSC therapies faces persistent challenges, including cell safety concerns, limitations in large-scale manufacturing, and inconsistencies in quality control and standardization. In contrast, MSC-derived exosomes (MSC-Exos) retain the therapeutic potential of their parental cells while offering enhanced advantages, such as reduced infusion-related toxicity, minimal immunogenicity, and superior tissue penetration.

Recently, the efficacy of MSC-Exos has been extensively studied and validated. MSC-Exos transport bioactive cargoes such as lipids, active enzymes, mRNAs, and microRNAs (miRNAs) to the recipient cells, mediating intercellular communication to regulate physiological processes and tissue homeostasis [5]. Among these components, miRNAs stand out as pivotal regulators due to their capacity to fine-tune gene expression networks. Notably, the exosomal miRNA profile is highly dynamic and influenced by variations in MSC culture conditions [6,7] and tissue sources [8,9]. The production consistency of exosomal miRNAs is depends on multiple factors, including culture systems, cell passage number, tissue source, and harvest timing [10,11].

Therefore, elucidating the specific exosomal miRNA profile and its regulatory targets is critical for uncovering their therapeutic potential and enabling precise clinical applications. Such investigations will facilitate the development of standardized, cell-free therapies with optimized efficacy and safety profiles, accelerating clinical adoption.

In this study, we established an optimized serum-free culture system for hUC-MSCs and determined the critical harvest window for exosomes. Using our optimized serum-free culture system and high-throughput sequencing, miR-16-5p was identified as the predominant miRNA that functions as a core immunosuppressive effector, significantly suppressing LPS/IFN-γ-induced M1 macrophage polarization and Th1 cell differentiation. Furthermore, miR-125b-5p and miR-34a-5p act in collaboration with miR-16-5p, orchestrating a multilayered regulatory network that simultaneously targets key nodes in the NF-κB and JAK-STAT pathways across both innate and adaptive immune systems. These findings provide novel insights into inflammation resolution strategies.

## 2 Materials and methods

### 2.1 Cell culture

HUC-MSCs were isolated from human umbilical cord specimens using established protocol as previously described [[12], [13], [14]]. In brief, hUC-MSCs were cultured in α-MEM medium (Gibco, #2357140) supplemented with 10 % fetal bovine serum (PAN Biotech, #ST200303), 100 U/ml penicillin, and 100 mg/ml streptomycin, under standard culture conditions (37 °C, 5 % CO_2_ humidified atmosphere). HUC-MSCs at passages 4–6 were subsequently characterized through analyses detailed in Supplementary 1. The study protocols were approved by Beijing Institute of Radiation Medicine ethics committee (AF/SC-08/02.255).

### 2.2 Isolation of exosomes and extraction and sequencing of exosomal miRNA

HUC-MSCs at passage 4–6 were cultured in improved α-MEM purchased from Tianjin Haoyang Biological Products Technology Co., Ltd, # SC2013-G for 24 h interval. Subsequently, the supernatant was collected at 24-, 48-, and 72-h for exosome isolation and identification. In brief, the culture medium was centrifuged to remove dead cells and cell debris and processed using the QIAGEN exosome isolation kit (#76064) for exosomes isolation [15]. Then the exosomal RNA was extracted using miRNeasy Micro Kit (QIAGEN 217084) and subjected to high-throughput sequencing on the Illumina NovaSeq X Plus platform.

### 2.3 mRNA determination via quantitative real-time PCR

Total RNA for qRT-PCR was extracted from cultured RAW264.7 and induced Th1 cells. The qRT-PCR primers were designed as Supplementary 4-Table 1. Briefly, 1–2 μg of total RNA was reverse-transcribed to cDNA using MMLV reverse transcriptase (Takara Biomedical Technology (Beijing) Co., Ltd) with random primers. The reverse-transcribed cDNA was performed real-time PCR via TaqMan PCR kit on an Applied Biosystems 7300 Sequence Detection System (Applied Biosystems).

### 2.4 M1 macrophage induction

The miRNA mimics or NC mimics (Supplementary 5-Table2) were complexed with JetPRIME reagent (# 101000027, Polyplus) and transfected into RAW264.7 cells at a final concentration of 0.2 μM. 12 h post-transfection, M1 macrophage was induced by adding LPS (#L2880, Sigma-Aldrich, 100 ng/ml) and murine IFN-γ (#AF-315-05, PeproTech, 10 ng/ml) to the culture medium. The transfected cells were then cultured in DMEM medium supplemented with 10 % fetal bovine serum at 37 °C and 5 % CO_2_ for additional 24 h prior to collection for further analysis.

### 2.5 Th1 cell differentiation

Naïve CD4^+^ T cells were isolated from murine spleen (Supplementary 7A) using the Naive CD4^+^ T Cell Isolation Kit (#130-104-453, Miltenyi Biotec) method and seeded at a concentration of 2 × 10^6^ cells/ml in RPMI 1640 culture medium (#22400105, Gibco). The medium was supplemented with recombinant human interleukin-2 (IL-2, #212–12, PeproTech, 10 ng/ml), interleukin-12 (IL-12, #210–12, PeproTech, 10 ng/ml), and anti-mouse-interleukin-4 (*anti*-IL-4, #81112–25, PeproTech, 1 μg/ml) to induce Th1 cell differentiation. Seventy-two hours post-induction, the cells were collected for further analysis.

### 2.6 Flow cytometry analysis

The M1 macrophage induced RAW264.7 cells or induced Th1 cells were harvested and washed with PBS. The single cells were labeled with iNOS (APC-iNOS, #17592082, eBioscience), F4/80 (FITC-F4/80, #35-4801-U100, Tonbo Biosciences), CD11b (PerCP-Cyanine5.5-CD11b, 65-0112-U100, Tonbo Biosciences), CD45(PE-CD45, #147712, Biolegend), CD3 (FITC-CD3, #35-0032-U100, Tonbo Biosciences) and CD4 (PE-CD4, #50-0041-U100, Tonbo Biosciences) respectively. The cells were fixed and permeabilized using the BD IntraSure Kit (#641776) and labeled with IFN-γ (APC-IFN-γ, #20-7311-U100, Tonbo Biosciences).

### 2.7 Luciferase activity assay

The wild-type 3′UTR and specific mutant 3′UTR of *mIFN-γ*, *mNOS2*, *mIRF1*, *mSTAT1* and *mNFKB1* were cloned into pmirGLO and co-transfected with miR-16-5p mimics or miR-125b-5p or miR-34a-5p to HELA cells, respectively. 48 h post transfections, the firefly luciferase activities and Renilla luciferase activities were measured by a Dual-Luciferase Reporter Assay System (#E1980, Promega).

## 3 Result

1 Exosomal miRNA profiling of hUC-MSCs identifies miR-16-5p as the predominant miRNA and core immunosuppressive effector

**Fig. 1:**
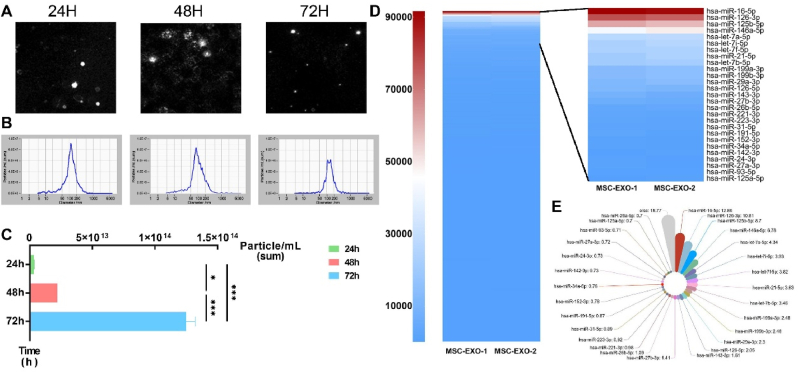
Exosomal miRNA profiling identifies the immunomodulatory effectors of MSC.

To explore the immunoregulatory effectors within MSC-Exos, we established an optimized serum-free culture system for human umbilical cord-derived MSCs (hUC-MSCs). Exosomes were sequentially isolated from conditioned culture supernatants at 24, 48, and 72 h, respectively (Fig. 1A). Nanoparticle tracking analysis (NTA) confirmed that the extracellular vesicles exhibited characteristic size distribution ranging from 50 nm to 150 nm, consistent with exosomal parameters (Fig. 1B).

Quantitative NTA revealed a temporal progression in exosome secretion, with maximal secretion predominantly between 48 and 72 h of culture (Fig. 1C). Based on this kinetic profile, culture supernatants were harvested after 72 h for exosome isolation and downstream miRNA analysis. Exosomal RNA was extracted using miRNeasy Micro Kit reagent followed by high-throughput sequencing on the Illumina NovaSeq X Plus platform.

Bioinformatic analysis identified 118 unique miRNAs with detectable expression levels (expression count >10 in all replicates), among which 28 highly abundant miRNAs (counts per million [CPM] >4000), constituting the top 23.7 %, collectively accounted for 81 % of total miRNA reads (Fig. 1D and E). These findings strongly suggest that these miRNAs may serve as principal immunoregulatory effectors in hUC-MSC-derived exosomes (hUC-MSC-Exos).

A: NTA was employed to determine the particle size of exosomes derived from hUC-MSCs; B: NTA analysis revealed that the size distribution of exosomes ranges from 50 nm to 150 nm; C: Quantitative NTA exhibit the production and secretion of exosomes at 24 h, 48 h, and 72 h of culture; D: Bioinformatics analysis identified 118 unique miRNAs with detectable expression levels (expression count>10 in both replicates), of which 28 miRNAs showed significantly increased expression (CPM>4000); E: The percentage of the 28 highly abundant miRNAs was determined, providing insights into their relative expression levels within the exosomal miRNA profile.

2 Exosomal miR-16-5p predominantly Suppress M1 Macrophage Polarization and Th1 Cell Differentiation

Comprehensive analysis of the exosomal miRNA atlas identified miR-16-5p as the predominant component among the 28 highly abundant miRNAs, accounting for 12.86 % of total exosomal miRNA content. Notably, functional enrichment analysis via miRPathDB revealed significant overrepresentation of miR-16-5p targets (*p* < 0.05) in immunomodulatory pathways, such as T cell activation, Jak-STAT signaling, and T cell receptor signaling (Supplementary 6-Table3). The abundant exosomal miR-16-5p and its close association with immune regulatory networks suggest that miR-16-5p plays a pivotal role in implementing the immunoregulatory effects of the MSCs.

**Fig. 2:**
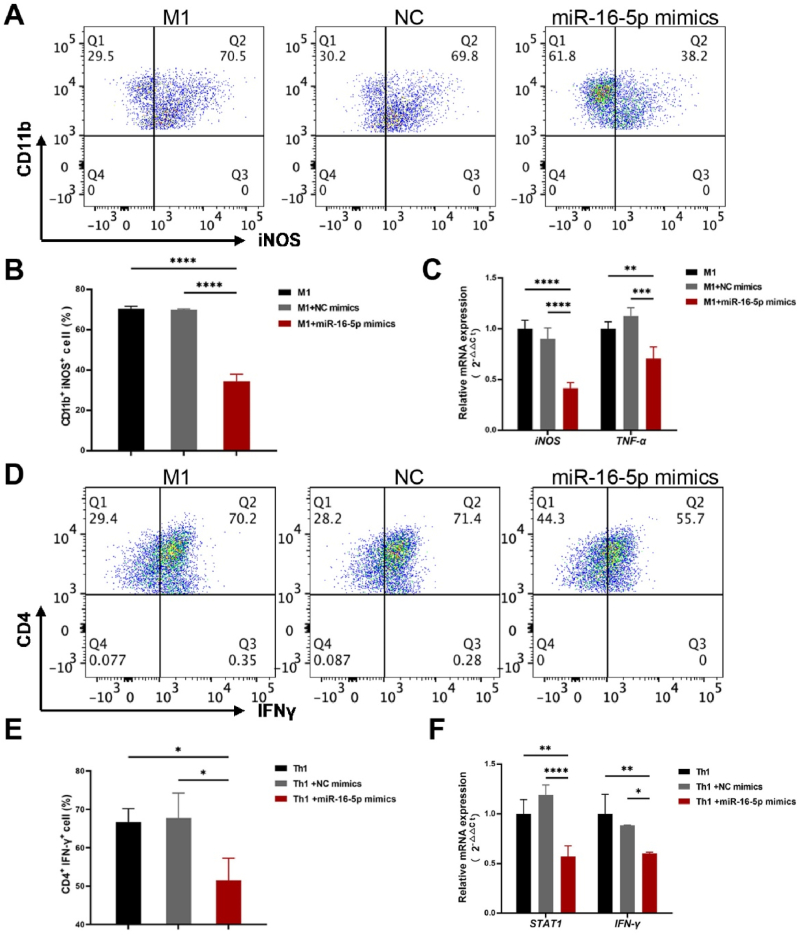
miR-16-5p mimics blocks M1 macrophage polarization and Th1 cell differentiation. A: Raw264.7 cells were transfected with miR-16-5p mimics or NC mimics and subsequently induced to M1 macrophage polarization. After 24 h of culture, the percentage of CD11b^+^ iNOS^+^ cells in each group were measured by flow cytometry. B: Data are presented as mean ± standard deviation ($\bar{x}\pm$ s), with n = 3. *p* < 0.0001 compared with M1 group and NC mimics group. C: Naïve CD4^+^ T cells were induced to Th1 cells and then transfected with miR-16-5p or NC mimic. After 5 days of culture, the percentage of CD4^+^ IFN-γ^+^ cells in each group were measured by flow cytometry. D: Data are presented as mean ± standard deviation ($\bar{x}$ ±s), with n = 3. *p* = 0.0319, compared with Th1 group *p* = 0.0237, compared with NC mimics group. E: The inhibitory effect of miR-16-5p transfection on the mRNA levels of iNOS and TNF-α in M1 cells was measured by q-PCR. Data are presented as mean ± standard deviation ($\bar{x}$ ±s), with n = 3. *p*_*iNOS*_<0.0001, *p*_*TNF-α*_ = 0.0038, compared with M1 group, *p*_*iNOS*_<0.0001, *p*_*TNF-α*_ = 0.0002, compared with NC mimics group. F: The mRNA expression levels of IFN-γ and STAT4 in different groups were measured by q-PCR. Data are presented as mean ± standard deviation ($\bar{x}$ ±s), with n = 3. *p*_*STAT1*_ = 0.0018, *p*_*I*_-_*γ*_ = 0.0032, compared with Th1 group, *p*_*STAT1*_ <0.0001, *p*_*I*_-_*γ*_ = 0.0286, compared with NC mimics group.

LPS/IFN-γ-induced M1 polarization was used to investigate the role of miR-16-5p in macrophage polarization. Flow cytometry analysis revealed that transfection with miR-16-5p mimics significantly attenuated M1 macrophage polarization, reducing the M1 population to 32.70 ± 3.39 % compared to 70.05 ± 0.38 % in the negative control group (NC; *p* < 0.0001) and 70.4 ± 1.2 % in the induced group (M1; *p* < 0.0001; Fig. 2A and B). Moreover, miR-16-5p mimics transfection markedly suppressed the expression of inducible nitric oxide synthase (iNOS) and tumor necrosis factor-alpha (TNF-α), as demonstrated by qRT-PCR (Fig. 2C). These findings collectively demonstrate that miR-16-5p serves as a critical suppressor in regulating macrophage polarization dynamics.

To further determine the immunosuppression effects of miR-16-5p, we induced Th1 polarization in vitro, and found that miR-16-5p mimics transfection disrupted Th1 cell polarization dynamics, resulting in a significant reduction of Th1 cell proportion from 67.77 ± 6.47 % in negative control group (NC) and 66.65 ± 3.55 % in the induced group (Th1) to 44.85 ± 9.65 % (*p*_NC_<0.05, *p*_Th1_<0.05, Fig. 2D and E). Besides, qRT-PCR results showed that miR-16-5p mimics transfection suppressed the expression levels of STAT1 and IFN-γ, the master transcriptional regulator and key cytokine of Th1 polarization. These findings further demonstrate that miR-16-5p suppresses Th1 polarization dynamics and serves as a critical immunoregulatory effectors in hUC-MSC-Exos.

3 Follow-up characterization of miR-125b-5p and miR-34a-5p in macrophage and Th1 cell regulation guided by miR-16-5p target prediction

To elucidate the molecular mechanism underlying miR-16-5p-mediated suppression of M1 macrophage polarization and Th1 differentiation, we performed an integrated bioinformatics prediction analysis followed by experimental validation. Initial computational screening identified evolutionarily conserved binding sites and specific mutations for miR-16-5p in the 3′UTRs of *NFKB1, IRF1, IFN-γ, STAT1,* and *NOS2* (Suppl. 2A-B), genes encoding critical components of the NF-κB and JAK/STAT signaling cascades. As shown in Suppl. 2C, co-transfection of miR-16-5p down regulated the expression of *NFKB1, IRF1, IFN-γ, STAT1,* and *NOS2* according to dual-luciferase reporter assays demonstrated miR-16-5p suppress the expression of NFKB1 (30 % reduction, *p*_*NFKB1*_<0.05), IRF1 (40 % reduction, *p*_IRF1_<0.05), *IFN-γ* (54 % reduction, *p*_IFN__-γ_<0.05)*, STAT1* (38 % reduction, *p*_*STAT1*_<0.001)*,* and *NOS2* (60 % reduction, *p*_*NOS2*_<0.01), respectively. These findings demonstrate that miR-16-5p's ability to regulate multiple nodes within these immunoregulatory pathways. This multi-targeting pattern suggests that miR-16-5p functions as a master regulator of inflammatory signaling through concerted suppression of both transcriptional regulators (IRF1, STAT1) and effector molecules (IFN-γ, NOS2).

Building on our identification of miR-16-5p as the principal immunomodulatory component of hUC-MSC-Exos, we speculated that additional miRNA-mediated suppression of inflammatory signaling pathways may be involved. Systematic bioinformatics prediction and analysis revealed miR-34a-5p and miR-125b-5p as synergistic partners in targeting NF-κB and JAK/STAT network components. Evolutionary conserved miR-125b-5p binding sites were identified in the 3′UTRs of *IFN-γ* and *IRF1* (Suppl. 3A), Dual-luciferase reporter assays demonstrated miR-125b-5p significantly downregulates the expression of IFN-γ (46 % reduction, *p*_IFN__-γ_ <0.001) and IRF1 (43 % reduction, *p*_IRF1_<0.0001) (Suppl. 3B), while miR-34a-5p exhibited complementary sequences in the 3′UTRs of *NFKB1*, *IRF1*, and *STAT1* (Suppl. 3C). Dual-luciferase reporter assays demonstrated miR-34a-5p suppress the expression of NFKB1 (20 % reduction, *p*_*NFKB1*_<0.0001), IRF1 (44 % reduction, *p*_IRF1_<0.0001), and STAT1 (24 % reduction, *p*_*STAT1*_<0.0001), respectively (Suppl. 3D). This coordinated targeting pattern reveals a collaborative miRNA network that simultaneously inhibits both upstream transcriptional regulators (STAT1, IRF1) and downstream inflammatory effectors (NFKB1, IFN-γ), establishing multi-layered regulation of pro-inflammatory signaling cascades.

**Fig. 3:**
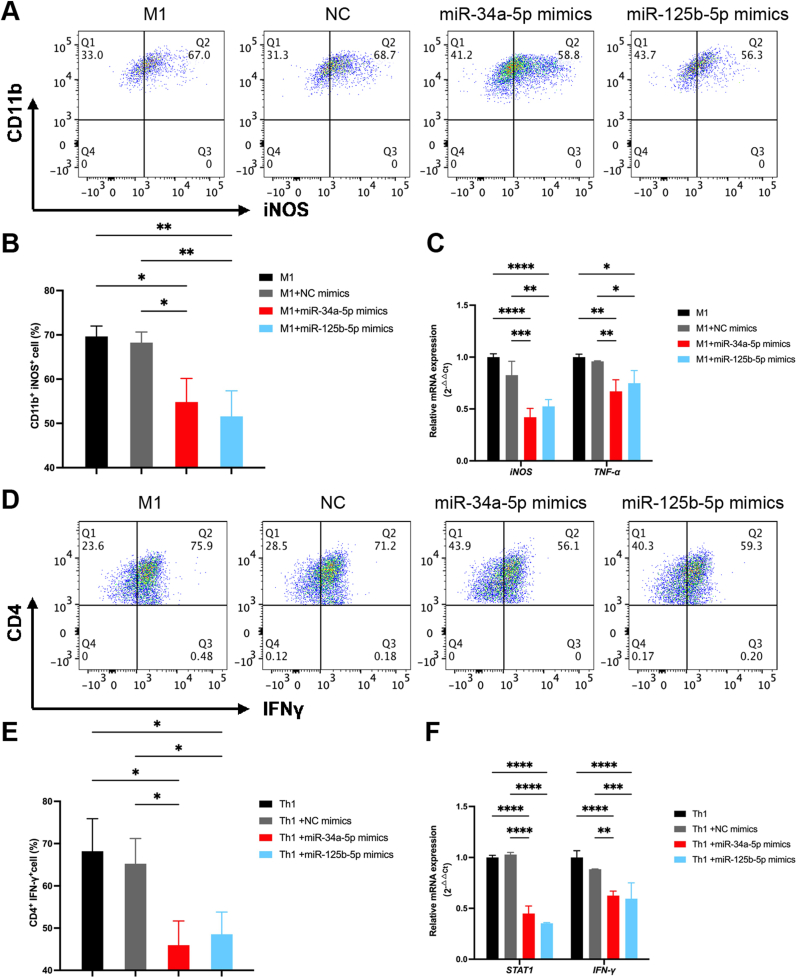
miR-34a-5p and miR-125b-5p mimics suppress M1 macrophage polarization and Th1 cell differentiation. A: The Raw264.7 cells were transfected miR-34a-5p mimic, miR-125b-5p mimics or NC mimics and subsequently induced to M1 macrophage. After 24 h of culture, the percentage of CD11b^+^ iNOS^+^ cells in various group was measured by flow cytometry. B: Data are presented as mean ± standard deviation ($\bar{x}\pm$ s), with n = 3. *p*_M1,34_ = 0.0118, *p*_M1,125_ = 0.0037, compared with M1 group; *p*_NC,34_ = 0.0202, *p*_NC,125_ = 0.0060, compared with NC mimics group. C: Naïve CD4^+^ T cells were induced to Th1 cells and then transfected with miR-34a-5p mimics, miR-125b-5p mimics or NC mimic. After 5 days of culture, the percentage of CD4^+^ IFN-γ^+^ cells in various groups was measured by flow cytometry. D: Data are presented as mean ± standard deviation ($\bar{x}\pm$ s), with n = 3. *p*_Th1,34_ = 0.0102, *p*_Th1,125_ = 0.0201, compared with Th1 group, *p*_NC,34_ = 0.0220, *p*_NC,125_ = 0.0447, compared with NC mimics group. E: The inhibitory effects of miR-34a-5p mimics or miR-125b-5p mimics transfection on the mRNA levels of *iNOS* and *TNF-α* in M1 cells was measured by q-PCR. Data are presented as mean ± standard deviation ($\bar{x}\pm$ s), with n = 3. *p*_34-iNOS_ <0.0001, *p*_125-iNOS_<0.0001, *p*_34-TNF-α_ = 0.0012, *p*_125-TNF-α_ = 0.0117, compared with M1 group, *p*_34-iNOS_ = 0.0001, *p*_125-iNOS_ = 0.0027, *p*_34-TNF-α_ = 0.0039, *p*_125-TNF-α_ = 0.0377, compared with NC mimics group. F: The mRNA expression level of *I*-*γ* and *STAT1* in different groups were measured by q-PCR. $\bar{x}\pm$ s, with n = 3. *p*_34-STAT1_ <0.0001, *p*_125-STAT1_<0.0001, *p*_3_-_γ_ <0.0001, *p*_1_-_γ_ <0.0001, compared with Th1 group, *p*_34-STAT1_ <0.0001, *p*_125-STAT1_<0.0001, *p*_3_-_γ_ = 0.0014, *p*_1_-_γ_ = 0.0004, compared with NC mimics group.

Flow cytometry analysis revealed that transfection with miR-34a-5p mimics or miR-125b-5p mimics significantly attenuated LPS/IFN-γ-induced M1 macrophage polarization, reducing the M1 population to 57.85 ± 5.31 % and 54.8 ± 5.8 %, compared to 69.55 ± 2.38 % in the NC group (*p* < 0.05; *p* < 0.01) and 69 ± 2.31 % in the M1 group (*p* < 0.01; *p* < 0.01; Fig. 3A and B). Moreover, miR-34a-5p mimics or miR-125b-5p mimics transfection significantly suppressed the expression of iNOS and TNF-α, as demonstrated by qRT-PCR (Fig. 3C). Parallel analysis in Th1 differentiation revealed a concerted immunomodulatory effect, with both mimics reducing Th1 cell proportion from 65.25 ± 5.95 % in NC group to 45.95 ± 5.75 % and 48.55 ± 5.25 % (*p* < 0.05; *p* < 0.05; Fig. 3D and E). In addition, qRT-PCR confirmed that both mimics transfection suppressed the expression level of STAT1 and IFN-γ (Fig. 3F), further supporting that miR-34a-5p and miR-125b-5p suppress Th1 polarization dynamics and serves as a critical immunoregulatory effectors in hUC-MSC-Exos.

## 4 Discussion

Since their first discovery in 1976 by Friedenstein and his colleagues, mesenchymal stem cells have gone through five decades of intensive scientific research from evaluating the therapeutic feasibility, biosafety profile and clinical efficacy to clinical trials and application today. MSCs are multipotent stromal cells with robust immunomodulatory [1], regenerative [2,3], and anti-inflammatory properties [16,17]. Studies have shown that MSCs could be successfully isolated from diverse tissues [7]. However, significant challenges remain in clinical translation, particularly regarding donor-dependent heterogeneity, tissue-specific functional differences, and the lack of standardized production criteria for therapeutic-grade cell products [18]. Their therapeutic effects are primarily mediated through paracrine rather than direct engraftment. The bioactive molecules secreted by MSCs, including growth factors, cytokines, and extracellular vehicles (EVs), modulate immune responses and promote tissue repair [19,20]. Preclinical and clinical studies highlight their efficacy in treating autoimmune diseases [21,22], inflammatory conditions, and degenerative disorders [23,24].

While the therapeutic efficacy of MSC-derived exosomes/EVs is well-established, exosomal miRNA consistency depends on multiple factors, including culture systems, cell passage number, tissue source, and harvest timing. In this study, we focused specifically on optimizing culture conditions and defining the critical harvest window for exosomes. Using our optimized serum-free culture system, miR-16-5p was identified as the predominant miRNA that functions as a core immunosuppressive effector, significantly suppressing LPS/IFN-γ-induced M1 macrophage polarization and Th1 cell differentiation. Notably, hUC-MSC-derived exosomal miR-125b-5p and miR-34a-5p collaborate with miR-16-5p to orchestrate a multilayered regulatory network that simultaneously target key nodes in the NF-κB and JAK-STAT pathways across both innate and adaptive immune systems.

MSC-derived exosomal miRNAs represent a promising cell-free therapeutic approach for immune modulation [25]. These miRNAs exhibit therapeutic effects by suppressing pro-inflammatory M1 macrophage polarization [26] and inhibiting Th1 cell differentiation [18], thereby attenuating hyperactive immune responses through NF-κB and JAK-STAT signaling pathways [3,20,[27], [28], [29]]. Current research on exosomes and EVs is primarily focused on enhancing production consistency, understanding pharmacokinetics, and overcoming clinical translation barriers to fully realize their therapeutic potential in treating immune-related disorders.

Our recent study found that individual mimics of miR-16-5p, miR-125b-5p and miR-34a-5p effectively suppress M1 macrophage polarization and Th1 differentiation. However, MSC-Exos naturally function as a complex cocktail of multiple miRNAs during biological processes, suggesting that the overall effects of all the components within the exosomes should be taken into account. Importantly, the relative abundance of each miRNA within the exosomes critically determines the overall functional outcome.

This engineered miRNA framework redefines MSC-Exos as dynamically programmable “immune nanoswitches”, capable of context-dependent modulation of inflammatory microenvironments. By precisely calibrating miRNA ratios and delivery kinetics, we would achieve unprecedented control over immune cell fate decisions-suppressing destructive Th1/M1 polarization while amplifying regenerative pathways, thereby addressing the critical unmet need for targeted immunomodulation in conditions ranging from autoimmune diseases to transplant rejection.

## CRediT authorship contribution statement

**Yuanjing Zheng:** Writing – original draft, Data curation, Conceptualization. **Yue Li:** Investigation, Data curation. **Zhengyang Wei:** Investigation. **Yang Wang:** Investigation. **Yuanlin Liu:** Methodology. **Fengsong Liu:** Writing – review & editing, Supervision, Project administration. **Xue Li:** Writing – review & editing, Writing – original draft, Project administration, Investigation, Data curation, Conceptualization. **Yi Zhang:** Writing – review & editing, Validation, Supervision.

## Ethical approval

The study protocols were approved by Beijing Institute of Radiation Medicine ethics committee (AF/SC-08/02.255).

## Declaration of competing interest

The authors declare that they have no competing interests and confirm that no commercial or financial relationships existed during this research that might pose a conflict of interest.

## Data Availability

Data will be made available on request.
