# Supplementary material for: HUC-MSC-derived exosomal miR-16-5p attenuates inflammation via dual suppression of M1 macrophage polarization and Th1 differentiation
Source: Biochem Biophys Rep. 2025 Jun 9;43:102078. doi: 10.1016/j.bbrep.2025.102078 (PMC12181010; doi:10.1016/j.bbrep.2025.102078)
Supplement: Multimedia component 1 [file mmc1.docx]

**Figure The biological characteristics of hUC-MSCs**. A: Detection of Mycoplasma in MSCs culture using Loop-mediated Isothermal Amplification (LAMP) method. NC: negative control; MSC1: sample 1; MSC2: sample 2; MSC3: sample 3; PC: positive control; B: Morphology of hUC-MSCs at passage 4; C: The cell surface markers of MSCs at passage 4 were detected via flow cytometry; D: Alkaline phosphatase (ALP) staining of hUC-MSCs following 10 days of osteogenic induction; E-F: The mRNA expression level and protein level of ALP and DLX5 following 10 days of osteogenic induction; G: Oil Red O staining of hUC-MSCs following 14 days of adipogenic induction; H-I: The mRNA expression level and protein level of PPAR-γ and C/EBP-α following 14 days of adipogenic induction. Data are presented as mean ± standard deviation (x̄±s), with n=3. *p_ALP_* =0.0003, *p_DLX5_* =0.0059, *p_PPAR-γ_* =0.0306, *p_C/EBP-α_* =0.0007, compared with the control group.
