# Supplementary material for: HUC-MSC-derived exosomal miR-16-5p attenuates inflammation via dual suppression of M1 macrophage polarization and Th1 differentiation
Source: Biochem Biophys Rep. 2025 Jun 9;43:102078. doi: 10.1016/j.bbrep.2025.102078 (PMC12181010; doi:10.1016/j.bbrep.2025.102078)
Supplement: Multimedia component 2 [file mmc2.docx]

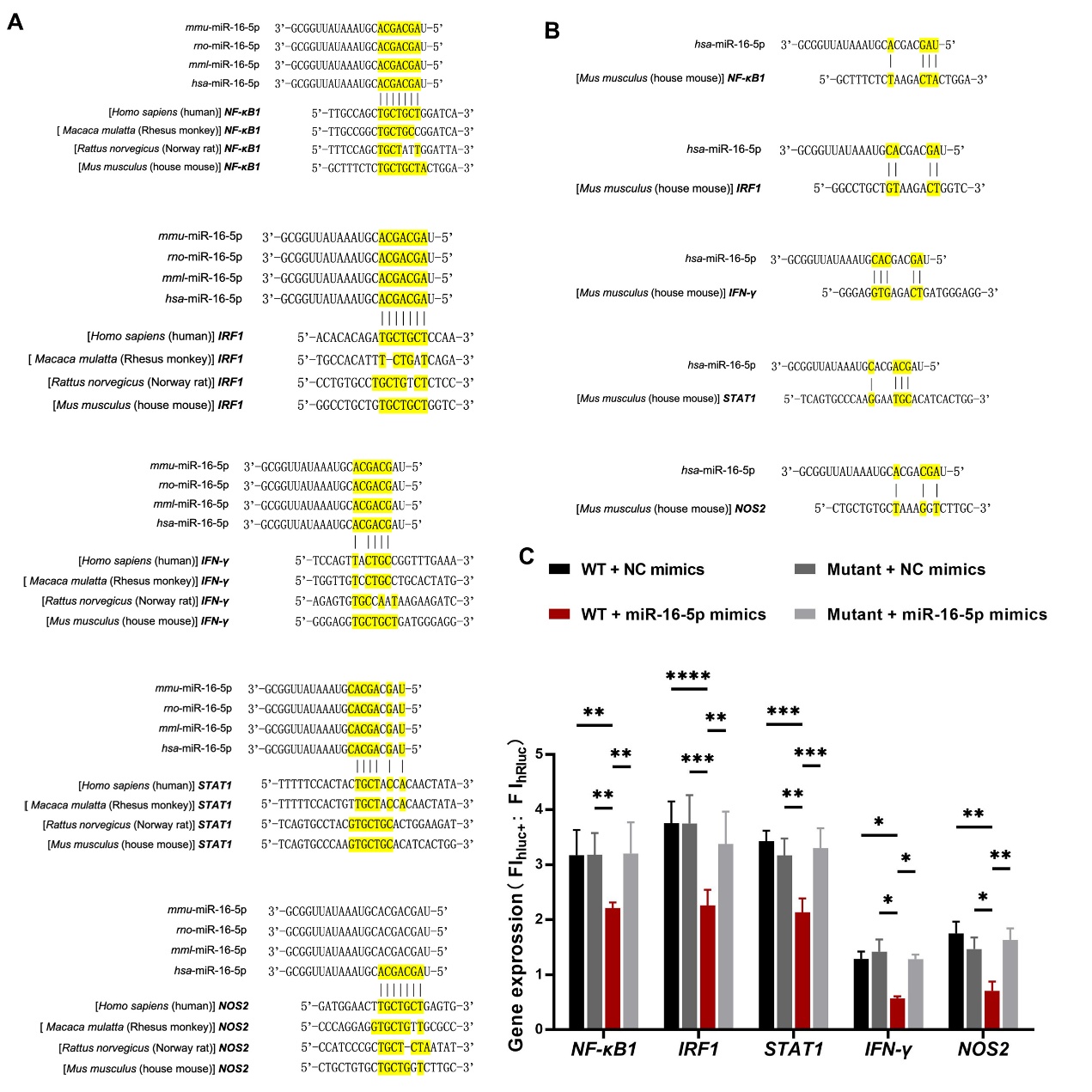


**Supplementary 2 Targeting sites of miR-16a-5p on 3′UTR of *NF-κB1, IRF1, STAT1, IFN-γ* and *NOS2*.**

The target sequence (A) and the mutant sequence (B) of *NF-κB1, IRF1, STAT1, IFN-γ* and *NOS2* were inserted into the 3′UTR of *hluc+* in pmirGLO vector respectively; C: the hluc+ expression was regulated by the target sequence. The hRluc expression was control. The fluorecent intensity（FI）ratio of *hluc+* to *hRluc* reported the expression levels of of *NF-κB1, IRF1, STAT1, IFN-γ* and *NOS2*. Co-transfection of miR-16-5p mimics downregulated the luciferase activity of hluc+ with 3′-UTR of WT-*NF-κB1*, WT- *IRF1*, WT-S*TAT1*, WT-*IFN-γ* and WT-*NOS2*, but did not affect the luciferase activity of hluc+ with 3′-UTR of MUT- *NF-κB1,* MUT- I*RF1*, MUT- *STAT1*, MUT-*IFN-γ* and MUT-*NOS2*. x̄±s, n=3. *p*_NFKB1_=0.0076, *p*_IRF1_=0.0023, *p*_IFNG_ =0.0408, *p*_STAT1_=0.0003, *p*_NOS2_ =0.0049, compared with NC mimics group with WT.
