# Supplementary material for: HUC-MSC-derived exosomal miR-16-5p attenuates inflammation via dual suppression of M1 macrophage polarization and Th1 differentiation
Source: Biochem Biophys Rep. 2025 Jun 9;43:102078. doi: 10.1016/j.bbrep.2025.102078 (PMC12181010; doi:10.1016/j.bbrep.2025.102078)
Supplement: Multimedia component 3 [file mmc3.docx]

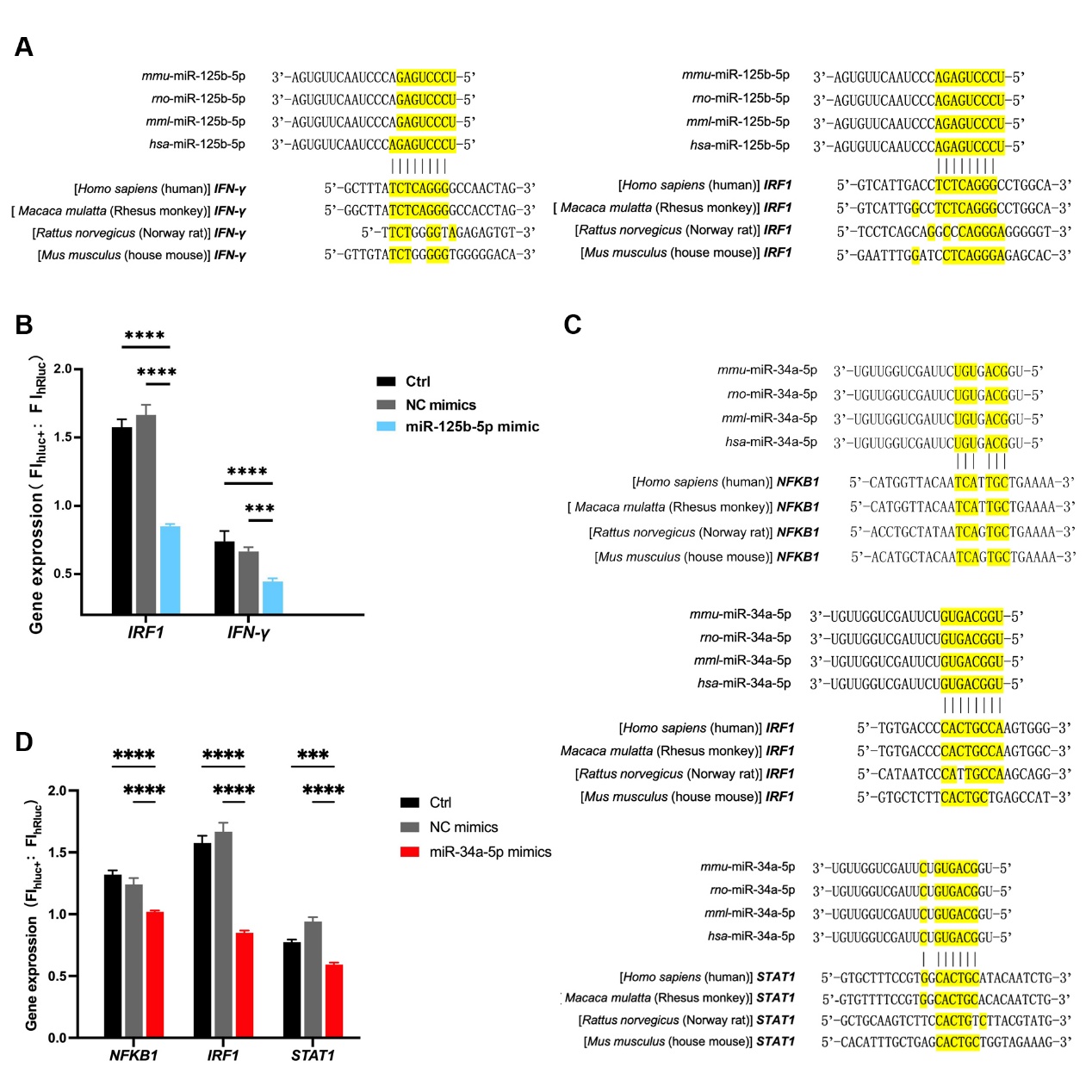


**Supplementary 3 Targeting sites of miR-34a-5p and miR-125b-5p on 3′UTR of *NFKB1, IRF1, STAT1* and *IFN-γ.***

A: Evolutionary conserved miR-125b-5p binding sites were identified in the 3′UTRs of *IFN-γ* and *IRF1*. B: Co-transfection of miR-125b-5p mimics downregulated the luciferase activity of *hluc+* with 3′-UTR of *IRF1* and *IFN-γ.* x̄±s, n=3. *p*_125-IRF1_< 0.0001, *p*_125-IFN-_*_γ_*< 0.0001, compared with control group, *p*_NC-IRF1_< 0.0001, *p*_NC-IFN-_*_γ_*=0.0006, compared with NC mimics group. C: Evolutionary conserved miR-34a-5p exhibited complementary sequences in the 3′UTRs of *NFKB1, IRF1,* and *STAT1*. D: Co-transfection of miR-34a-5p mimics downregulated the luciferase activity of hluc+ with 3′-UTR of *NFKB1, IRF1 and STAT1.* x̄±s, n=3. *p*_34-NFKB1_< 0.0001, *p*_34-IRF1_< 0.0001, *p*_34-STAT1_= 0.0001, compared with control group, *p*_NC-NFKB1_< 0.0001, *p*_NC-IRF1_ < 0.0001, *p*_NC-STAT1_= < 0.0001, compared with NC mimics group*.*
