# Supplementary material for: HUC-MSC-derived exosomal miR-16-5p attenuates inflammation via dual suppression of M1 macrophage polarization and Th1 differentiation
Source: Biochem Biophys Rep. 2025 Jun 9;43:102078. doi: 10.1016/j.bbrep.2025.102078 (PMC12181010; doi:10.1016/j.bbrep.2025.102078)
Supplement: Multimedia component 4 [file mmc4.docx]

| **Table 1 Primer sequences for q-PCR** | |
| --- | --- |
| **GENE** | **Sequence (5’-3’)** |
| GAPDH | Forward：ACTCTTCCACCTTCGATGC |
|  | Reverse：CCGTATTCATTGTCATACCAGG |
| iNOS | Forward：ACTCTTCCACCTTCGATGC |
|  | Reverse：CCGTATTCATTGTCATACCAGG |
| TNFα | Forward：ACTCTTCCACCTTCGATGC |
|  | Reverse：CCGTATTCATTGTCATACCAGG |
| IFN-γ | Forward：ATGAACGCTACACACTGCATC |
|  | Reverse：CCATCCTTTTGCCAGTTCCTC |
| STAT1 | Forward：GCTGCCTATGATGTCTCGTTT |
|  | Reverse：TGCTTTTCCGTATGTTGTGCT |
