# Supplementary material for: HUC-MSC-derived exosomal miR-16-5p attenuates inflammation via dual suppression of M1 macrophage polarization and Th1 differentiation
Source: Biochem Biophys Rep. 2025 Jun 9;43:102078. doi: 10.1016/j.bbrep.2025.102078 (PMC12181010; doi:10.1016/j.bbrep.2025.102078)
Supplement: Multimedia component 5 [file mmc5.docx]

| **Table 2 Sequence of microRNA mimics** | |
| --- | --- |
| **miRNA mimics** | **Sequence (5’-3’)** |
| NC mimic | Sense：rUrGrArGrArArCrUrGrArArUrUrCrCrArUrGrGrGrUrU |
|  | Antisense：mC*mC*mCmAmUmGmGmAmAmUmUmCmAmGmUmUmCmUmC*mA*mU*mU* |
| hsa-miR-16-5p mimic | Sense：rUrArGrCrArGrCrArCrGrUrArArArUrArUrUrGrGrCrG |
|  | Antisense：mC*mC*mAmAmUmAmUmUmUmAmCmGmUmGmCmUmGmCmU*mA*mU*mU* |
| hsa-miR-34a-5p mimic | Sense：rUrGrGrCrArGrUrGrUrCrUrUrArGrCrUrGrGrUrUrGrU |
|  | Antisense：mA*mA*mCmCmAmGmCmUmAmAmGmAmCmAmCmUmGmCmC*mA*mU*mU* |
| hsa-miR-125b-5p mimic | Sense：rUrCrCrCrUrGrArGrArCrCrCrUrArArCrUrUrGrUrGrA |
|  | Antisense：mA*mC*mAmAmGmUmUmAmGmGmGmUmCmUmCmAmGmGmG*mA*mU*mU* |
