# Supplementary material for: HUC-MSC-derived exosomal miR-16-5p attenuates inflammation via dual suppression of M1 macrophage polarization and Th1 differentiation
Source: Biochem Biophys Rep. 2025 Jun 9;43:102078. doi: 10.1016/j.bbrep.2025.102078 (PMC12181010; doi:10.1016/j.bbrep.2025.102078)
Supplement: Multimedia component 6 [file mmc6.docx]

**Table 3 functional enrichment analysis of miR-16-5p targets via miRPathDB**

| **Database** | **Pathway** | **Evidence** | **Hits** | **Expected hits** | **P-value** | **Targets** |
| --- | --- | --- | --- | --- | --- | --- |
| KEGG | Jak-STAT signaling pathway | experimental (strong) | 8 | 1.801 | 0.002 | AKT3, CCND1, CCND2, CCND3, IFNG, IL12B, PIM1, SOCS3 |
| Gene Ontology - Biological Process | T cell lineage commitment | experimental (strong) | 4 | 0.414 | 0.011 | BCL2, IL12B,MTOR,TP53 |
| Gene Ontology - Biological Process | positive T cell selection | experimental (strong) | 3 | 0.414 | 0.045 | BCL2, IL12B,MTOR |
| Gene Ontology - Biological Process | positive regulation of CD4-positive, alpha-beta T cell activation | experimental (strong) | 3 | 0.414 | 0.045 | IFNG, IL12B,MYB |
| Gene Ontology - Biological Process | CD4-positive or CD8-positive, alpha-beta T cell lineage commitment | experimental (strong) | 3 | 0.292 | 0.026 | BCL2, IL12B,MTOR |
| Gene Ontology - Biological Process | alpha-beta T cell lineage commitment | experimental (strong) | 3 | 0.292 | 0.026 | BCL2, IL12B,MTOR |
| Gene Ontology - Biological Process | T cell activation involved in immune response | experimental (strong) | 5 | 1.071 | 0.033 | IFNG, IL12B,MTOR,MYB,TP53 |
| Gene Ontology - Biological Process | alpha-beta T cell activation | experimental (strong) | 6 | 1.606 | 0.036 | ADORA2A, BCL2, IFNG, IL12B, MTOR, MYB |
| Gene Ontology - Biological Process | T cell differentiation | experimental (strong) | 8 | 2.726 | 0.038 | BCL2, CDK6, IFNG, IL12B, MTOR, MYB, TP53, WNT4 |
| Gene Ontology - Biological Process | T cell activation | experimental (strong) | 11 | 4.721 | 0.04 | ADORA2A, BCL2, CCND3, CDK6, IFNG, IL12B, MTOR, MYB, NCSTN, TP53, WNT4 |
| Gene Ontology - Biological Process | positive regulation of CD4-positive, alpha-beta T cell differentiation | experimental (strong) | 3 | 0.389 | 0.04 | IFNG, IL12B,MYB |
| Reactome | Gene and protein expression by JAK-STAT signaling after Interleukin-12 stimulation | experimental (any) | 10 | 3.639 | 0.02 | HNRNPA2B1, HNRNPDL, HNRNPF, HSPA9, IFNG, PAK2, PDCD4, RPLP0, SNRPA1, TCP1 |
| Reactome | TRAF6 mediated induction of NFkB and MAP kinases upon TLR7/8 or 9 activation | predicted (intersection) | 39 | 22.85 | 0.005 | APP,BTRC,CHUK,CREB1,DUSP3,FBXW11,IRAK2,IRAK4,MAP2K1,MAP2K3,MAP2K6,MAP2K7,MAPK1,MAPK10,MAPK11,MAPK14,MAPK3,MAPK8,MAPK9,MAPKAPK2,MEF2C,NFKB1,NKIRAS1,NOD1,PELI2,PELI3,PPP2CA,PPP2R1A,PPP2R1B,PPP2R5D,RPS6KA3,SKP1,TAB1,TAB3,TLR7,TNIP2,TRAF6,UBA52,UBE2V1 |
| KEGG | T cell receptor signaling pathway | predicted (intersection) | 42 | 25 | 0.004 | AKT2,BCL10,CARD11,CBLC,CD28,CD3D,CD3E,CD4,CDC42,CHUK,DLG1,FYN,GSK3B,ICOS,ITK,KRAS,MAP2K1,MAP2K7,MAPK1,MAPK11,MAPK14,MAPK3,MAPK9,NFATC2,NFATC3,NFKB1,PAK1,PAK2,PAK3,PAK6,PDCD1,PDPK1,PIK3CB,PIK3R1,PIK3R2,PIK3R3,PPP3CB,RAF1,RHOA,SOS2,TEC,VAV2 |
| KEGG | T cell receptor signaling pathway | predicted (union) | 63 | 44.96 | 0.002 | AKT2, BCL10, CARD11, CBL, CBLB, CBLC, CD28, CD3D, CD3E, CD4, CD8B, CDC42, CHUK, CSF2, DLG1, FOS, FYN, GRAP2, GSK3B, HRAS, ICOS, ITK, KRAS, LAT, MAP2K1, MAP2K2, MAP2K7, MAP3K7, MAPK1, MAPK11, MAPK13, MAPK14, MAPK3, MAPK9, NFATC1, NFATC2, NFATC3, NFKB1, NFKBIB, PAK1, PAK2, PAK3, PAK4, PAK6, PDCD1, PDPK1, PIK3CA, PIK3CB, PIK3CG, PIK3R1, PIK3R2, PIK3R3, PIK3R5, PLCG1, PPP3CB, PPP3CC, PPP3R1, RAF1, RHOA, SOS2, TEC, VAV2, VAV3 |
| WikiPathways | RANKL/RANK (Receptor activator of NFKB (ligand)) Signaling Pathway | predicted (union) | 36 | 24.17 | 0.011 | AKT2, CBL, CDC42, CHUK, FHL2, FOS, GAB2, LYN, MAP2K1, MAP2K6, MAP2K7, MAP3K7, MAPK1, MAPK14, MAPK3, MAPK8, MAPK9, MITF, NFATC1, NFKB1, PAPSS2, PIK3R1, PIK3R2, PLCG1, SQSTM1, SRC, STAT1, SYK, TAB1, TNFRSF11A, TRAF1, TRAF2, TRAF3, TRAF5, TRAF6, VCAM1 |
