# Supplementary material for: HUC-MSC-derived exosomal miR-16-5p attenuates inflammation via dual suppression of M1 macrophage polarization and Th1 differentiation
Source: Biochem Biophys Rep. 2025 Jun 9;43:102078. doi: 10.1016/j.bbrep.2025.102078 (PMC12181010; doi:10.1016/j.bbrep.2025.102078)
Supplement: Multimedia component 7 [file mmc7.docx]

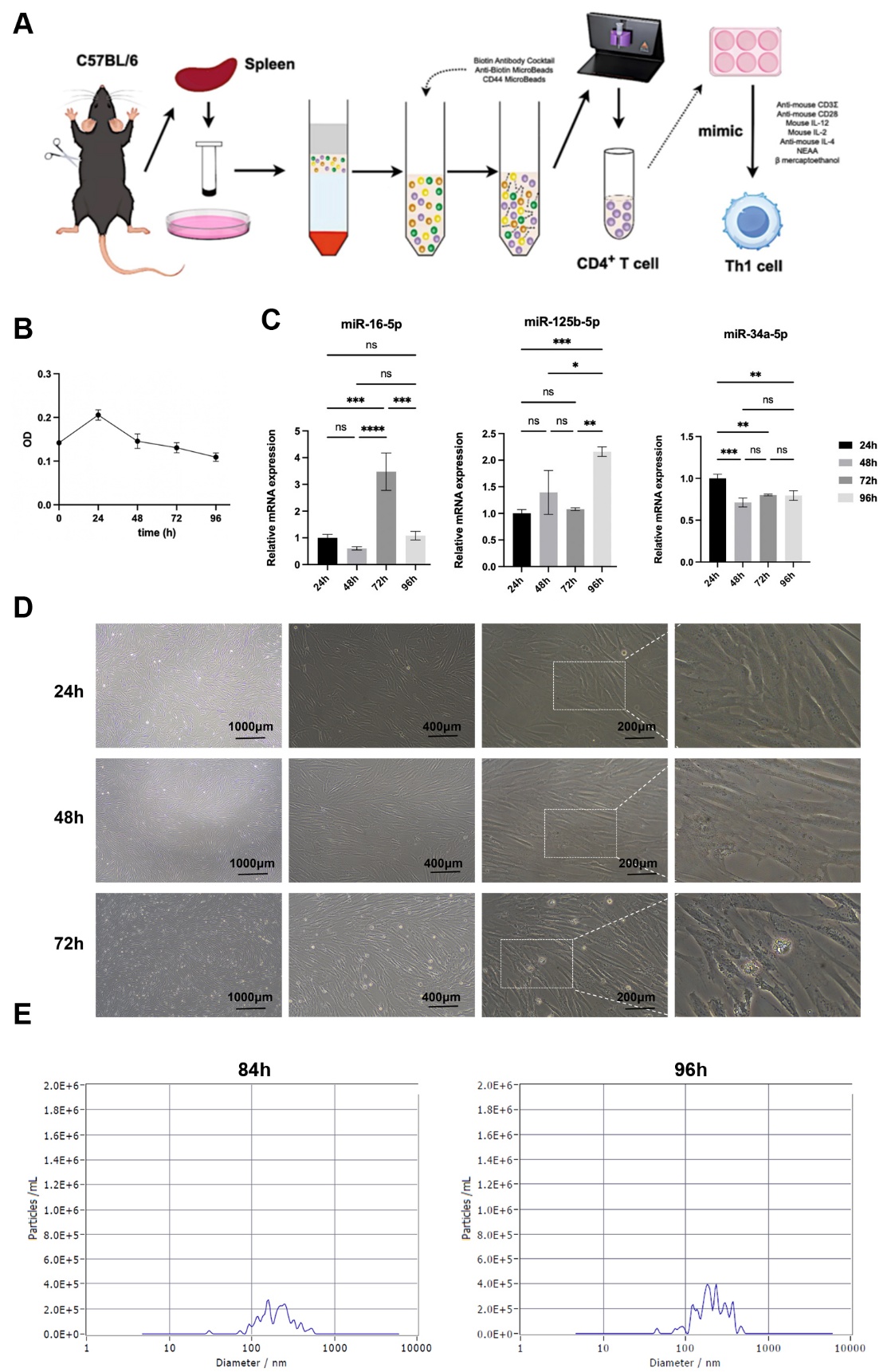


**Supplementary 7 Isolation of naive CD4^+^ T cells, miRNA expression profiling, hUC-MSC culture, and exosome characterization.**

**(A)** Schematic representation of naive CD4^+^ T cell isolation from murine spleen. Naïve CD4^+^ T cells were isolated from murine spleen using the Naive CD4^+^ T Cell Isolation Kit (#130-104-453, Miltenyi Biotec) method and seeded at a concentration of 2×10^6^ cells/ml in RPMI 1640 culture medium (#22400105, Gibco). The medium was supplemented with recombinant human interleukin-2 (IL-2, #212-12, PeproTech, 10 ng/ml), interleukin-12 (IL-12, #210-12, PeproTech, 10 ng/ml), and anti-mouse-interleukin-4 (anti-IL-4, #81112-25, PeproTech, 1μg/ml) to induce Th1 cell differentiation. Seventy-two hours post-induction, the cells were collected for further analysis.

**(B)** Cell viability and proliferation were evaluated using the Cell Counting Kit-8 (CCK-8) assay. Conditioned human umbilical cord mesenchymal stem cells (hUC-MSCs) were seeded at a density of 5 × 10⁵ cells per 75 cm² culture flask.

**(C)** Relative quantification of exosomal miR-16-5p, miR-125-5p, and miR-34a-5p at 0-24-hour, 24-48-hour, 48-72-hour and 72-96-hour intervals.

**(D)** Morphological assessment of hUC-MSCs under conditional culture.

**(E)** Nanoparticle tracking analysis (NTA) of exosomes derived from hUC-MSCs at 72-84-hour and 84-96-hour culturing.
